# Dynamic Functional Connectivity Reveals Abnormal Variability in the Amygdala Subregions of Children With Attention-Deficit/Hyperactivity Disorder

**DOI:** 10.3389/fnins.2021.648143

**Published:** 2021-09-29

**Authors:** Yue Yang, Binrang Yang, Linlin Zhang, Gang Peng, Diangang Fang

**Affiliations:** Children’s Healthcare & Mental Health Center, Shenzhen Children’s Hospital, Shenzhen, China

**Keywords:** ADHD, dynamic functional connectivity, anxiety, amygdala, rs-fMRI

## Abstract

**Objective:** This study investigates whether the dynamic functional connectivity (dFC) of the amygdala subregions is altered in children with attention-deficit/hyperactivity disorder (ADHD).

**Methods:** The dFC of the amygdala subregions was systematically calculated using a sliding time window method, for 75 children with ADHD and 20 healthy control (HC) children.

**Results:** Compared with the HC group, the right superficial amygdala exhibited significantly higher dFC with the right prefrontal cortex, the left precuneus, and the left post-central gyrus for children in the ADHD group. The dFC of the amygdala subregions showed a negative association with the cognitive functions of children in the ADHD group.

**Conclusion:** Functional connectivity of the amygdala subregions is more unstable among children with ADHD. In demonstrating an association between the stability of functional connectivity of the amygdala and cognitive functions, this study may contribute by providing a new direction for investigating the internal mechanism of ADHD.

## Introduction

Globally, nearly 1 in 20 youth suffers from attention-deficit/hyperactivity disorder (ADHD), making it one of the most prevalent psychiatric disorders among children (Polanczyk et al., 2007; Polanczyk et al., 2014). Inattention, hyperactivity, and impulsivity are the three recognized core symptoms of ADHD (American Psychiatric Association, 2013); however, research deems these symptoms inadequate to explain the functional impairment of children (Anastopoulos et al., 2011; Skirrow and Asherson, 2013; Szuromi et al., 2013). Research on school-age children revealed that most children with ADHD have psychiatric comorbidities that worsen functional impairments (Cuffe et al., 2020). This condition includes markedly elevated rates of anxiety disorders, with common features such as excessive fear, anxiety, and avoidance behavior.

Studies on anxiety over the last few decades have further clarified the impairment associated with ADHD (Prevatt et al., 2015; Overgaard et al., 2016; D’Agati et al., 2019). Abundant evidence suggests that people with ADHD have elevated levels of anxiety. For instance, researchers found that up to 25% of children with ADHD have comorbid anxiety disorders (Wolraich et al., 2005). O’Rourke et al. (2020) believe that people with ADHD are at a greater risk of having anxiety symptoms and its associated features than people without ADHD. Similarly, Gau et al. (2010) noticed that children and adolescents with persistent ADHD have a higher risk of developing anxiety disorders than those without persistent ADHD. People suffering from ADHD and with high levels of anxiety have demonstrated significantly different performances than individuals with pure ADHD in terms of working memory deficits, symptoms of impulsivity, and cognitive efficiency (Schatz and Rostain, 2006; Sorensen et al., 2011; Prevatt et al., 2015). For example, a recent meta-analysis suggested that patients with both ADHD and an anxiety disorder had better response inhibition, compared with individuals afflicted only with ADHD (Maric et al., 2018). March et al. (2000) found that children with ADHD and anxiety exhibited more symptoms of inattentiveness than impulsivity. In a recent study, the researchers found that adolescents with higher trait anxiety performed better on indices of sustained attention, reaction time, and motor variability among people with ADHD but not among those without ADHD (Ruf et al., 2017).

It is important to study the complex mechanisms underlying ADHD, together with the functional impairments of the diseased brain, to ensure effective diagnosis, treatment, and prevention. Resting-state functional magnetic resonance imaging (rs-fMRI) is a safe and non-invasive tool that can detect spontaneous activity in the brain (Bin et al., 2018). The amygdala, which can respond to a wide range of emotional stimuli in time, has long been considered a critical component of emotional processing (Aghajani et al., 2014; Fox et al., 2015). In the study of pediatric anxiety, the amygdala is also the most frequently examined region of interest (ROI) (Hamm et al., 2014). The association between the functional connectivity (FC) of the amygdala and elevated anxiety levels has been identified in numerous clinical populations via this technology (Gold et al., 2016; Jalbrzikowski et al., 2017; Porta-Casteras et al., 2020). For example, Jalbrzikowski et al. (2017) specified that increased FC based on the centromedial amygdala (CMA)–rostral anterior cingulate cortex is associated with greater anxiety symptoms during early adulthood, whereas increased structural connectivity in the CMA–anterior ventromedial prefrontal cortex (PFC) white matter is associated with greater anxiety during late childhood (Jalbrzikowski et al., 2017). In another study involving children aged 7 to 9 years, researchers found that high childhood anxiety is associated with abnormal function and volume of the amygdala (Qin et al., 2014). The amygdala has also been found to contribute to cognitive functions such as working memory and executive functions (Schaefer et al., 2006; Schaefer and Gray, 2007). Previous studies have proven that the amygdala of patients with ADHD is significantly abnormal in terms of function and volume compared with those of normal people (Yu et al., 2016; Tajima-Pozo et al., 2018). Hence, the relationship among the amygdala, anxiety, and cognitive functions may provide essential insights into the psychopathology of ADHD. However, the amygdala is a complex structure and may functionally segregate into several subregions (Han et al., 2014). According to an rs-fMRI study, the FC patterns of each of the amygdala subregions in healthy humans is distinctive (Roy et al., 2009). Thus far, there have been few similar studies on children with ADHD.

Reliance on the implicit assumption that participants’ brain activity remained static throughout the rs-fMRI scan was a common feature of many previously mentioned studies. However, a growing body of research confirms that brain activity changes dynamically over time (Yao et al., 2017; Liao et al., 2019; Li et al., 2019). Most rs-fMRI studies in the ADHD domain currently focus on the characteristics of the static state of brain activity but fail to demonstrate the dynamic temporal changes in spontaneous brain activity among humans. Using the sliding window approach, researchers have inspected the dynamic mechanisms of voluntary brain activity in humans (Voytek and Knight, 2015) and non-human primates (Hutchison et al., 2013). The abnormalities concerning the variance of dynamic FC (dFC) in patients with common neuropsychiatric diseases, such as schizophrenia (Guo et al., 2018), Alzheimer’s disease (Gu et al., 2020), major depressive disorders (Yao et al., 2019), and autism spectrum disorder (Li et al., 2020), have been effectively revealed by the sliding window method. These studies suggest that changes in dFC may be biological markers of specific diseases. In this approach, a fixed-length time window is selected and used to calculate the FC metric. The window then slides to the next time window after a predetermined duration, leading to many FC metrics that can elucidate the temporal features of FC over the entire duration of the scan (Yue et al., 2018). By calculating the time-varying covariance of interregional neural signals, dFC can describe precisely the collaboration of brain regions; that is, the higher the value of the dFC, the more unstable the FC (Yao et al., 2017). Therefore, further investigation of the overall dFC between brain regions may be necessary. In this regard, several studies have found abnormal dFC in ADHD patients. For example, a recent study found that patients with ADHD had significantly changed dFC of the cingulo-opercular network and sensorimotor network (Sun et al., 2021). Another research reported that the default-mode and task-positive networks in people with ADHD exhibit a quasi-periodic clustering recurrence pattern during the entire rs-fMRI scan, suggesting that dFC alterations in people with ADHD may be a neuroimaging marker for ADHD (Kaboodvand et al., 2020). However, no systematic study on the dynamic characteristics of abnormal amygdala-related neural networks in people with ADHD has emerged thus far.

To address this gap, we first used the sliding window method to study the abnormal dFC of the amygdala subregions of children with ADHD, specifically focusing on anxiety symptoms and cognitive functions, and further studied the relationship between the dFC of the amygdala subregions and anxiety symptoms and cognitive functions. Based on previous studies, we hypothesized that (1) higher dFCs of the amygdala subregions can be observed in children with ADHD; (2) higher dFC is positively associated with anxiety symptoms among children with ADHD; and (3) higher dFC is negatively associated with the cognitive functions of children with ADHD.

## Materials and Methods

### Participants

In total, 76 drug-naive ADHD boys aged 8 to 10 years were recruited from Shenzhen Children’s Hospital. All patients were interviewed by two experienced psychiatrists, fulfilling the diagnostic criteria for ADHD based on clinical interviews that follow the *Diagnostic and Statistical Manual of Mental Disorders, Fourth Edition*. The Schedule for Affective Disorder and Schizophrenia for School-Aged Children Present and Lifetime Version (K-SADS-PL) was used to interview the participants and their parents (Kaufman et al., 1997). Meanwhile, 20 boys aged 8 to 10 years were recruited from the Children’s Health Care Department of the same hospital for the healthy control (HC) group. Children in this group, together with their parents, were also interviewed using the K-SADS-PL to ensure that they did not meet the diagnosis of ADHD or any other mental disorders. Notably, all participants were Han Chinese.

The inclusion criteria also included normal vision and hearing and a Full-Scale Intelligence Quotient (FSIQ) ≥ 70 estimated by the Wechsler Intelligence Scale for Children, Fourth Edition. Participants who currently or previously had psychological disorders or who had serious physical disorders, neurological disorders, or brain injuries were excluded from this study. This study was approved by the Medical Research Ethics Committee of the Shenzhen Children’s Hospital. All children agreed to participate, and written informed consent was obtained from their parents.

### Measures of Anxiety Symptoms

Conner’s Parent Rating Scale (Conners et al., 1998) is a widely used scale for screening children’s behavioral problems, especially ADHD, and comprises items that assess anxiety. The scale’s Chinese version has good reliability and validity and is used to evaluate children between 3 and 16 years old (Conners et al., 1998). This scale constitutes five factors: behavioral problems, learning problems, psychosomatic disorders, hyperactivity impulse, and anxiety. This study chose four items to assess the anxiety symptoms of children, namely, “fear of new environments, places, new people, and going to school,” being “more afraid of loneliness, illness, or death than others,” “bashfulness,” and having a “feeling that he or she is threatened frequently.” The questionnaire, which was completed by the parents according to their observations of their child, adopted a four-level scoring method (0, 1, 2, and 3).

### Cognitive Measures

The neuropsychological test battery consisted of the Stroop color-word and N-back tests. The two tests were considered representative of two areas of cognitive function: response inhibition and working memory.

The Stroop test, translated from the Victorian version (Lee and Chan, 2010), consists of three cards printed with colors, representing color, word, and color–word tasks. The color task consists of colored dots; the word task comprises ordinary words that are unrelated to the meaning of color; the color–word task consists of words written in color that indicate the meaning of the color, but the color of these words differ from the meaning of the word itself. All these tasks require participants to read the color of dots or words and the researcher to record the time it takes participants to read through each card and the number of mistakes they make. Interference control ability is represented by the interference score (IS), that is, the time taken to complete the color–word task minus the time taken to complete the word–word task, or the number of errors made in the color–word task minus the number of errors made in the word–word task.

The N-back task consists of three subtasks, namely, 0-back, 1-back, and 2-back tasks. The 0-back task requires participants to press the left mouse button when a gray square appears at the top left of the central fixation (“ + ”) and to press the right mouse button when the square appears at the top right or right bottom of central fixation (“ + ”). The 1-back (2-back) task requires participants to identify whether the orientation of the X square displayed on the screen is the same as that of the X-1 (X-2) square. If the orientation is the same, they press the right mouse button; otherwise, they press the left button.

### rs-fMRI Data Acquisition

The rs-fMRI data for all participants were obtained using a 3.0-T system scanner (Siemens Magnetom Skyra), and all participants were instructed to close their eyes, relax, stay awake, and try not to move their head during the scan. Participants were checked after the scan to ensure that they remained awake during the procedure. The rs-fMRI data were obtained using an echo-planar imaging sequence with the following parameters: repetition time (TR) = 2,000 ms; echo time = 30 ms; flip angle = 90°; field of view = 220 × 220 mm^2^; matrix size = 94 × 94; 32 axial slices; and slice thickness = 3 mm and 130 volumes.

### Preprocessing

rs-fMRI data preprocessing was performed using the Data Processing and Analysis of Brain Imaging (DPABI) toolbox (http://rfmri.org/dpabi; Yan et al., 2016). The first 10 volumes were discarded to allow the machine to reach magnetization equilibrium and enable participants to adapt to the MRI scanning environment. The remaining volumes were processed based on the following steps: slice timing, head motion correction, normalization to a Montreal Neurological Institute template via the gray matter segment, and resampling to isotropic 3-mm voxels. Multiple nuisance covariates (i.e., the estimated motion parameters based on the Friston-24 model (Friston et al., 1996), the linear drift, the white matter signal, and the cerebrospinal fluid signal) were regressed out from the data. Finally, the rs-fMRI time series were temporal bandpass filtered (0.01 < *f* < 0.10 Hz).

### Head Motion

Following Jenkinson’s relative root-mean-square algorithm (Jenkinson et al., 2002), the mean framewise displacement (FD) generated during the scanning process was excluded. Notably, the participant was excluded if the mean FD exceeded 0.2 mm. Based on this criterion, one participant was excluded, and the study had a final total sample of 75 ADHD and 20 HC children. We used mean FD as a covariable in the subsequent statistical analysis to further reduce the impact of head movement on the findings.

### Dynamic Functional Connectivity Analysis

To examine whole-brain dFC, using a seed method based on the anatomical ROIs, we used six previously validated seed ROIs in the bilateral amygdala (Zimmermann et al., 2018). These regions were divided into the basolateral amygdala (BLA), CMA, and superficial amygdala (SFA; radius = 6 mm; the detailed coordinates of each seed are provided in Supplementary Table S1). The mean blood oxygen level–dependent (BOLD) time series of each seed region was extracted from the rs-fMRI data of each participant (Chao-Gan and Yu-Feng, 2010) and correlated with the BOLD time series of each voxel in the brain to generate six three-dimensional dFC maps.

A sliding window method was used in this study to formulate dFC maps for each participant. This method can elucidate the temporal features of FC over the entire duration of the scan and calculate the time-varying covariance of interregional neural signals, which is the variance of dFC. The window length is a key parameter based on the sliding window method. According to Leonardi and van de Ville (2015), the minimum window length should not be less than 1/*f*_min_ because a very short window length may cause spurious fluctuations. Additionally, the *f*_min_ denotes the minimum frequency of the time courses. Conversely, if a window length is too long, the dynamic characteristics of the time series would be rendered unobservable. Hence, we decided to use a Hamming window with a width of 50 TRs (100S) and a step of 1 TR. Moreover, the entire rs-fMRI time series was segmented into 71 windows for each participant. The resting-state *r* value matrix was obtained by computing the partial correlation coefficients, and the *z* value matrix was obtained via Fisher-*z* transformation. Before the statistical analyses, the result maps were smoothed out with a 4-mm full-width at the half-maximum Gaussian kernel. Furthermore, to exclude the influence of window width on the results, the window width was set as 32/64 TR to repeat the calculations. The results were similar with the results of the 50TR and are detailed in the Supplementary Materials Figure S1.

### Statistical Analyses

The Statistical Package for the Social Sciences 21.0 was used to analyze the demographic and clinical data. A two-sample *t* test was used to evaluate differences in age, grade, FSIQ, mean FD, anxiety scores, and cognitive scores between the ADHD and HC groups. Moreover, dFC maps were analyzed using the two-sample *t* test based on DPABI to distinguish dynamic changes in FC between the two groups. Previous studies based on rs-fMRI have shown that FC in the brain is significantly associated with intelligence (Song et al., 2008; Pamplona et al., 2015). Therefore, we included the FSIQ as a covariate, in addition to mean FD and age. The Gaussian random field theory was applied for multiple comparison corrections (two-tailed, voxel *p* < 0.001, cluster *p* < 0.05) in DPABI (Yan et al., 2016). Furthermore, we conducted partial correlation analyses between anxiety scores/cognitive scores and dFC, which showed significant difference between the groups in the two-sample *t* test, while using mean FD, age, and FSIQ as covariates, to examine the association between the dFC of the amygdala subregions and the anxiety symptoms and cognitive functions of children with ADHD. *p* < 0.05 was considered statistically significant. However, the two groups had an imbalanced number of participants, and this may have resulted in low statistical power. To test the reproducibility of this study’s results, we selected an equal number of children from the ADHD group as in the control group and rematched the children by age, grade, and FSIQ. The two-sample *t* tests were performed, and the same statistical method was used.

## Results

### Demographic Information

Altogether, 75 children with ADHD and 20 HC children were recruited to participate in this study, and their demographic and clinical information is provided in Table 1. A comparison between the two groups showed no statistical difference in age, education level, and psychosomatic disorders, whereas significant differences were observed in the mean FD, FSIQ, behavioral problems, learning problems, hyperactivity–impulse scores, anxiety scores, IS, and N-back scores of the two groups. As expected, the FSIQ and the N-back scores of the ADHD group were significantly lower than those of the HC group, whereas their behavioral problems, learning problems, hyperactivity–impulse scores, IS, and anxiety scores were higher.

**TABLE 1:** The participants’ demographic and clinical information.

	**ADHD (*n* = 75)**	**HC (*n* = 20)**	***p* values**
Age, mean ± SD (y)	8.86 ± 0.58	8.93 ± 0.68	0.75
Grade, mean ± SD	2.88 ± 0.73	2.63 ± 0.77	0.14
FSIQ, mean ± SD	86.07 ± 8.05	95.85 ± 10.20	<0.01
Mean FD, mean ± SD	0.07 ± 0.03	0.10 ± 0.06	0.01
Anxiety scores, mean ± SD	0.66 ± 0.54	0.14 ± 0.19	< 0.01
Behavioral problems, mean ± SD	1.14 ± 0.51	0.41 ± 0.35	< 0.01
Learning problems, mean ± SD	1.91 ± 0.63	0.57 ± 0.45	< 0.01
Psychosomatic disorders, mean ± SD	0.26 ± 0.31	0.23 ± 0.30	0.71
Hyperactivity–impulse scores, mean ± SD	1.59 ± 0.68	0.39 ± 0.43	< 0.01
IS(time), mean ± SD	19.43 ± 11.46	11.08 ± 4.00	< 0.01
IS(error), mean ± SD	2.22 ± 2.72	1.20 ± 1.61	0.04
Correct rate of working memory, mean ± SD			
0-back	0.87 ± 0.14	0.94 ± 0.08	0.01
1-back	0.63 ± 0.21	0.80 ± 0.17	< 0.01
2-back	0.41 ± 0.15	0.56 ± 0.14	< 0.01

*ADHD means attention-deficit/hyperactivity disorder; FD, framewise displacement; FSIQ, Full-Scale Intelligence Quotient; HC, health control; IS, interference score.*

### Group dFC Comparisons

**FIGURE 1:**
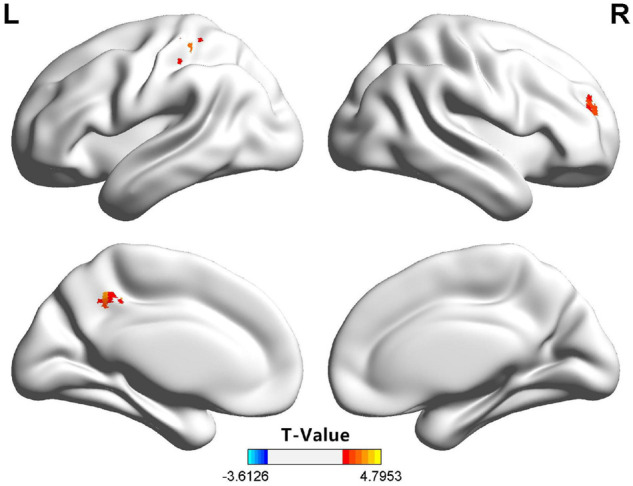
Compared with the HC group, the right SFA of the children in the ADHD group exhibited significantly higher dFC with the right PFC, the left precuneus, and the left post-central gyrus. ADHD, attention-deficit/hyperactivity disorder; dFC, dynamic functional connectivity; HC, health control; PFC, prefrontal cortex; SFA, superficial amygdala.

For the patients with ADHD (vs. the HC group), the dFC of the amygdala subregions showed significant differences in some areas, and the right SFA exhibited a significantly higher dFC in the right PFC, the left precuneus, and the left post-central gyrus. However, no regional differences in dFC were found between the children with ADHD and those in the HC group with regard to the bilateral BLA, CMA, and the left SFA. Detailed information is shown in Figure 1 and Table 2. After we rematched the subjects (Supplementary Materials Table S1), the results of the group dFC comparisons were slightly different (Supplementary Materials Figure S2). However, the dFC between the right SFA and the right PFC for the ADHD group was still higher than that for the HC group.

**TABLE 2:** Brain areas with significant dFC differences between children in the ADHD and HC groups.

**Regions**	**Brodmann areas**	**Voxels size**	**Peak MNI coordinates**			***t* value**
			***x***	***Y***	***z***	
Right PFC	10	45	30	48	24	3.49
Left precuneus	5/7/31	42	−12	−48	45	4.01
Left post-central gyrus	2/3/4	53	−24	−36	48	4.31

*ADHD means attention-deficit/hyperactivity disorder; HC, health control; MNI, Montreal Neurological Institute; PFC, prefrontal cortex; SFA, superficial amygdala.*

### Correlation Analysis

**FIGURE 2:**
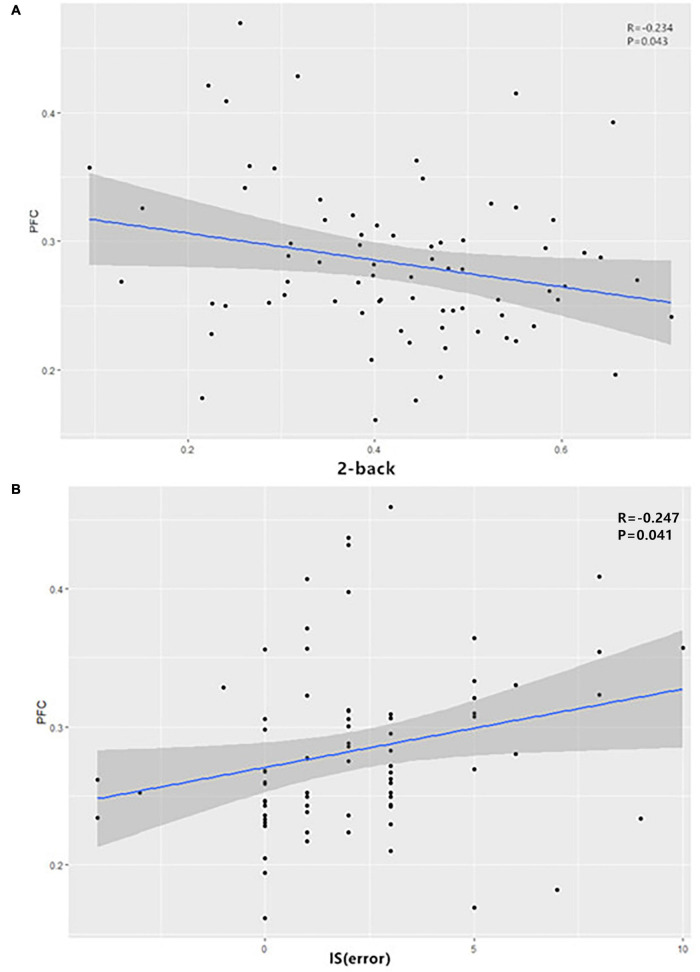
In ADHD patients, the dFC between the right SFA and the right PFC showed **(A)** a negative correlation with the 2-back scores and **(B)** a positive correlation with IS (error). ADHD, attention-deficit/hyperactivity disorder; dFC, dynamic functional connectivity; IS, interference score; SFA, superficial amygdala.

For patients with ADHD, the dFC between the right SFA and the right PFC showed a negative correlation with the 2-back scores (*r* = -0.234, *p* = 0.043) and a positive correlation with the IS (error) (*r* = −0.247, *p* = 0.041) (Figure 2). However, no significant results were found regarding the anxiety symptoms.

## Discussion

To our knowledge, this study is the first to use the dFC method to explore the amygdala subregion network of children with ADHD. We observed that compared with the HC group, the right SFA of the ADHD group showed a higher dFC; this highlights greater temporal variability in FC with the right PFC, the left precuneus, and the left postcentral gyrus. Another key finding is the correlation between the subregion of the amygdala and cognitive functions of patients with ADHD. The dFC of the amygdala subregions showed a specific association with working memory and response inhibition among patients with ADHD. These outcomes suggest that the FC of the amygdala is more unstable among children with ADHD, and the dFC of the amygdala subregion network is related to the cognitive functions of children with ADHD.

Unlike the resting-state FC, the stability of the FC is reflected by the dFC. The findings of this study may provide new insights into the abnormal brain activity of children with ADHD. Previous studies that analyzed the amygdala as a whole failed to recognize its structural complexity. Instead, research demonstrated that different subregions of the amygdala perform different functions (Han et al., 2014). Primarily, BLA is involved in associative learning processes as it receives incoming signals from the cortex and subcortical regions, including the PFC, thalamus, and hippocampus (Roy et al., 2009; Bzdok et al., 2013). Meanwhile, CMA is involved in attention regulation, motor generation, and autonomous emotional responses (Pessoa, 2011; Bzdok et al., 2013), whereas SFA addresses olfactory and reward-related information (Heimer and Van Hoesen, 2006; Bzdok et al., 2013).

In this study, the right SFA of the ADHD group showed a significantly higher FC variability in the right PFC, the left precuneus, and the left post-central gyrus. Previous studies suggested that the PFC is fundamentally involved in mechanisms underlying anxiety (Bishop, 2007; Hare et al., 2008). In the brain, the PFC and amygdala are interconnected and work in concert to control the expression of emotions, such as fear and anxiety (Likhtik et al., 2014). The PFC exerts inhibitory top-down control over amygdala activities under physiological conditions, preventing inappropriate emotional expressions (Rosenkranz et al., 2003). Several studies have shown that the PFC is necessary for the neurobiology of ADHD (Cheng et al., 2017; Chen et al., 2020; Liu et al., 2020). Posner et al. (2011) detected a stronger FC between the amygdala and PFC in adolescents with ADHD. In contrast to previous studies, our study further analyzed the dFC of the amygdala subregion, taking into account its heterogeneity. We analyzed the dFC of the amygdala, and the elevated dFC represented poor stability of FC; this means that the interaction between the amygdala and PFC is unstable in children with ADHD.

Generally, the precuneus belongs to the parietal lobe, which is involved in a variety of complex functions and is critical for emotion processing (Cavanna and Trimble, 2006), mediation of subjective happiness, and somatomotor processing (Sato et al., 2015). The amygdala is involved in several of these functions, including emotional processing. The results obtained in our study, along with the proven psychological functions of these regions, suggest that the unstable functional connection between the amygdala and the parietal lobe may be a potential cause of ADHD. A survey of adolescents with ADHD signified that there was significantly less activation in the parietal lobe when these adolescents were asked to perform a mental rotation task that required spatial working memory; this may indicate parietal dysfunction in patients with ADHD (Vance et al., 2007). Hale et al. (2015) believe that parietal electroencephalogram asymmetry is associated with mood and anxiety disorders. However, in our study, we did not find a relationship between anxiety symptoms and brain regions. This may be because (1) in this study, the factors from Conner’s Parent Rating Scale were used to represent anxiety symptoms, which had no correlation with the three brain regions; and (2) the sample size of this study is relatively small.

This study also finds a relationship between the dFC of the amygdala subregions and the cognitive functions of patients with ADHD. Specifically, for patients with ADHD, the dFC between the right SFA and the right PFC showed a negative correlation with the 2-back scores and a positive correlation with the IS (error). This means that the more unstable the functional connection between SFA and PFC, the worse the cognitive function of patients with ADHD. Halperin and Schulz (2006) believe that the structure and function of the PFC are intimately involved in the manifestation of ADHD symptoms. A previous study found a reduced activation in the left PFC of children with ADHD during an inhibition task (Miao et al., 2017). Among people with ADHD, an increasing number of studies have shown that there are functional changes in the PFC. For example, a study found that young people with ADHD show decreased FC of the left dorsolateral PFC for high-load visuospatial working memory (Bedard et al., 2014). Another study found that a higher brain signal variability in the medial prefrontal areas is related to overall ADHD symptom severity and inattention across children with an ADHD diagnosis (Nomi et al., 2018), which is consistent with our findings. However, a previous study showed that increased brain signal variability is associated with improved task performance (Garrett et al., 2013). This contradicts our results and might be explained by the Yerkes–Dodson curve. There is an optimal value for brain functioning. It is possible that the signal variability in the PFC of people with ADHD exceeds this optimal value and thus appears to impair cognitive performance.

This study’s outcomes should be interpreted with caution because of the following limitations. First, the scanning time was relatively short, which might reduce the reliability of the rs-fMRI data. Each participant underwent an rs-fMRI scan for 260 s, whereas similar studies were typically performed for 5 to 8 min (Yu et al., 2016). Second, several methods, with unverified consistencies, have been proposed to calculate the dFC. However, this study used the sliding time window method only to explore the differences in the amygdala subregion network of children with ADHD. Various methods can be used to confirm the results of this study in future work. Third, the number of participants in the two groups was imbalanced, and the sample size of this study was relatively small, resulting in low statistical power. This disparity arose because of (1) the difficulty of recruiting participants who were willing to provide experimental data and (2) the use of stringent inclusion and exclusion criteria. Randomized controlled trials with larger sample sizes are warranted in the future to verify the findings of this study.

Despite these limitations, the following two advantages enhance the reliability of the results. Previous studies have outlined that stimulants can change the brain structure and function of patients with ADHD (An et al., 2013; Querne et al., 2017; Walhovd et al., 2020) and the dose of the stimulant correlates with the size of the amygdala (Becker et al., 2015). The participants selected for this study were not on any stimulants. Furthermore, researchers confirmed differences in FC among children with ADHD belonging to different age groups (Tang et al., 2018). This study minimized the confounding effect of age by including children between 8 and 10 years old and controlling for the participants’ ages in the statistical analysis.

In summary, we investigated the dynamic variability of amygdala-based FC and its association with anxiety symptoms and the cognitive function of children with ADHD. The results of this study suggest that the dFC of the amygdala subregion of children with ADHD is significantly different from that of healthy children, and higher dFCs are negatively associated with the cognitive functions of children with ADHD. In demonstrating an association between the variability of amygdala FC and cognitive functions, this study may contribute by providing a new direction for studying the internal mechanism of ADHD.

## Data Availability Statement

The raw data supporting the conclusions of this article will be made available by the authors, without undue reservation.

## Ethics Statement

The studies involving human participants were reviewed and approved by Medical Research Ethics Committee of Shenzhen Children’s Hospital. Written informed consent to participate in this study was provided by the participants’ legal guardian/next of kin.

## Author Contributions

YY and BY: conceptualization. LZ and DF: methodology and investigation. YY, BY, and GP: validation. YY and GP: formal analysis. BY and GP: resources and writing—review and editing. YY and DF: data curation. YY, LZ, and GP: writing—original draft preparation. BY: project administration and funding acquisition. All authors discussed the results and reviewed the manuscript.

## Conflict of Interest

The authors declare that the research was conducted in the absence of any commercial or financial relationships that could be construed as a potential conflict of interest. The reviewer LS declared a past collaboration with one of the author BY to the handling editor.

## Publisher’s Note

All claims expressed in this article are solely those of the authors and do not necessarily represent those of their affiliated organizations, or those of the publisher, the editors and the reviewers. Any product that may be evaluated in this article, or claim that may be made by its manufacturer, is not guaranteed or endorsed by the publisher.

## Supplementary Material

Supplementary Figure S1 Brain areas with significant dFC differences between children in the ADHD and HC groups (the window width was set as 32/64 TR). ADHD, attention-deficit/hyperactivity disorder; HC, health control; TR, repetition time.

## Abbreviations

ADHD: attention-deficit/hyperactivity disorder
BLA: basolateral amygdala
BOLD: blood oxygen level-dependent
CMA: centromedial amygdala
dFC: dynamic functional connectivity
DPABI: Data Processing and Analysis of Brain Imaging
FC: functional connectivity
FD: framewise displacement
FSIQ: full-scale intelligence quotient
HC: health control
K-SADS-PL: Schizophrenia for School-Aged Children Present and Lifetime Version
IS: interference score
PFC: prefrontal cortex
ROIs: regions of interest
rs-fMRI: Resting-state functional magnetic resonance imaging
SFA: superficial amygdala
TR: repetition time.
