# Supplementary material for: Dynamic Functional Connectivity Reveals Abnormal Variability in the Amygdala Subregions of Children With Attention-Deficit/Hyperactivity Disorder
Source: Front Neurosci. 2021 Sep 29;15:648143. doi: 10.3389/fnins.2021.648143 (PMC8514188; doi:10.3389/fnins.2021.648143)
Supplement: Supplementary Figure S1 — Brain areas with significant dFC differences between children in the ADHD and HC groups (the window width was set as 32/64 TR). ADHD, attention-deficit/hyperactivity disorder; HC, health control; TR, repetition time. [file Data_Sheet_1.docx]

Table S1. The re-matched participants’ demographic and clinical information.

|  | ADHD (n = 20) | HC (n = 20) | P-values |
| --- | --- | --- | --- |
| Age (Mean ± SD) | 8.63±0.48 | 8.93±0.68 | 0.16 |
| Grade (Mean ± SD) | 2.90±0.72 | 2.63±0.77 | 0.21 |
| FSIQ (Mean ± SD) | 96.02±5.34 | 95.85±10.20 | 0.26 |
| Mean FD (Mean ± SD) | 0.07±0.01 | 0.10±0.06 | 0.82 |
| Anxiety scores (Mean ± SD) | 0.74±0.58 | 0.14±0.19 | <0.01 |
| Behavioral problems(Mean ± SD) | 1.04±0.55 | 0.41±0.35 | <0.01 |
| Learning problems(Mean ± SD) | 1.79±0.57 | 0.57±0.45 | <0.01 |
| Psychosomatic disorders(Mean ± SD) | 0.47±0.39 | 0.23±0.30 | 0.04 |
| Hyperactivity impulsescores (Mean ± SD) | 1.54±0.60 | 0.39±0.43 | <0.01 |
| IS (Time; Mean ± SD) | 17.91±9.56 | 11.08±4.00 | 0.02 |
| IS (Error; Mean ± SD) | 1.80±1.82 | 1.20±1.61 | 0.06 |
| Correct rate of working memory (Mean ± SD) |  |  |  |
| 0-back | 0.89±0.15 | 0.94±0.08 | 0.30 |
| 1-back | 0.60±0.21 | 0.80±0.17 | <0.01 |
| 2-back | 0.39±0.18 | 0.56±0.14 | <0.01 |

**Notes:** ADHD means attention deficit hyperactivity disorder; HC, health control; FSIQ, Full-Scale Intelligence Quotient; FD, framewise displacement; and IS, interference score.


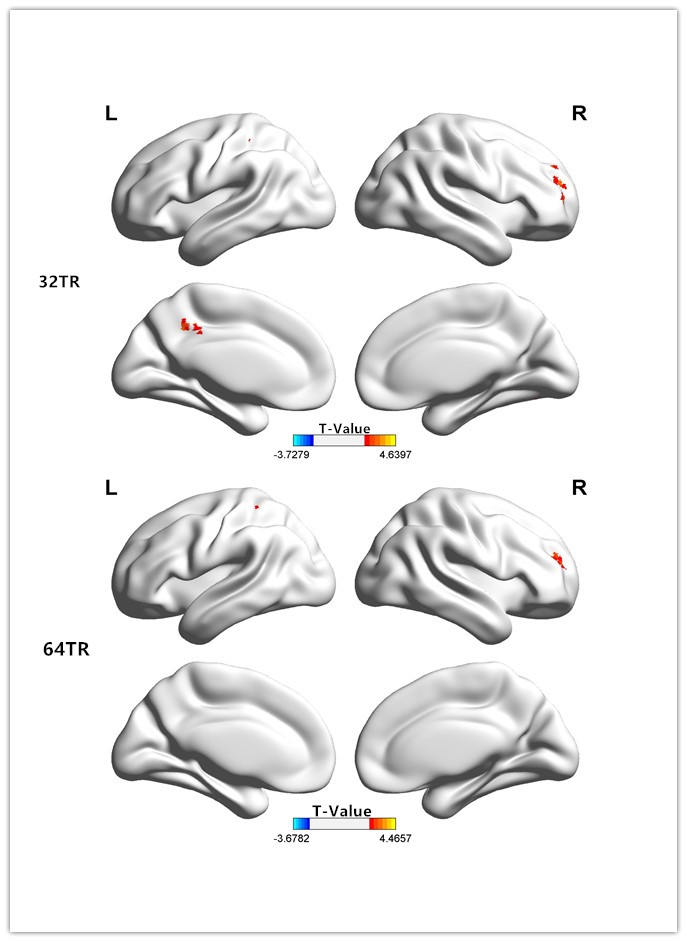


Figure S1. Brain areas with significant dFC differences between children in the ADHD and HC groups(The window width was set as 32/64 TR).

**Notes:**ADHD means attention deficit hyperactivity disorder; HC, health control; and TR,repetition time.


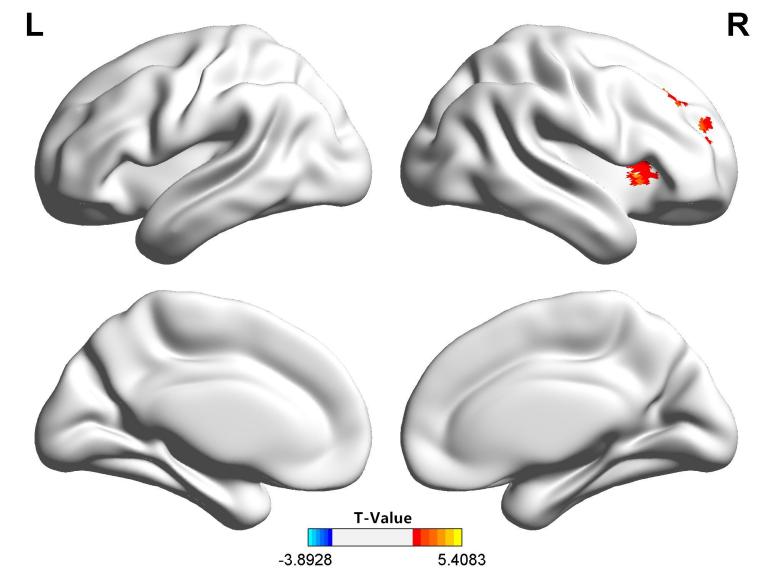


Figure S2. Compared with the HC group, the dFC in the right SFA of the children in the ADHD group (re-matched) was significantly higher.

**Notes:**ADHD means attention deficit hyperactivity disorder; HC, health control; and dFC, dynamic functional connectivity.
